# Asymmetric Alternative Current Electrochemical Method Coupled with Amidoxime-Functionalized Carbon Felt Electrode for Fast and Efficient Removal of Hexavalent Chromium from Wastewater

**DOI:** 10.3390/nano13050952

**Published:** 2023-03-06

**Authors:** Yunze Yang, Lun Lu, Yi Shen, Jun Wang, Liangzhong Li, Ruixue Ma, Zahid Ullah, Mingdeng Xiang, Yunjiang Yu

**Affiliations:** 1 Key Laboratory of Subsurface Hydrology and Ecological Effects in Arid Region, Ministry of Education, School of Water and Environment, Chang’an University, Xi’an 710064, China; 2 State Environmental Protection Key Laboratory of Environmental Pollution Health Risk Assessment, South China Institute of Environmental Sciences, Ministry of Ecology and Environment, Guangzhou 510655, China; 3 Key Laboratory of Microbial Technology for Industrial Pollution Control of Zhejiang Province, College of Environment, Zhejiang University of Technology, Hangzhou 310032, China; 4 State Key Laboratory of Separation Membranes and Membrane Processes, School of Environmental Science and Engineering, Tiangong University, Tianjin 300387, China; 5 State Key Laboratory of Biogeology and Environmental Geology, School of Environmental Studies, China University of Geosciences, Wuhan 430074, China

**Keywords:** alternating current electrochemistry, amidoxime-functionalized electrode, adsorption, reduction, Cr (VI) removal

## Abstract

A large amount of Cr (VI)-polluted wastewater produced in electroplating, dyeing and tanning industries seriously threatens water ecological security and human health. Due to the lack of high-performance electrodes and the coulomb repulsion between hexavalent chromium anion and cathode, the traditional DC-mediated electrochemical remediation technology possesses low Cr (VI) removal efficiency. Herein, by modifying commercial carbon felt (O-CF) with amidoxime groups, amidoxime-functionalized carbon felt electrodes (Ami-CF) with high adsorption affinity for Cr (VI) were prepared. Based on Ami-CF, an electrochemical flow-through system powered by asymmetric AC was constructed. The mechanism and influencing factors of efficient removal of Cr (VI) contaminated wastewater by an asymmetric AC electrochemical method coupling Ami-CF were studied. Scanning Electron Microscopy (SEM), Fourier Transform Infrared (FTIR), and X-ray photoelectron spectroscopy (XPS) characterization results showed that Ami-CF was successfully and uniformly loaded with amidoxime functional groups, and the adsorption capacity of Cr (VI) was more than 100 times higher than that of O-CF. In particular, the Coulomb repulsion effect and the side reaction of electrolytic water splitting were inhibited by the high-frequency anode and cathode switching (asymmetric AC), the mass transfer rate of Cr (VI) from electrode solution was increased, the reduction efficiency of Cr (VI) to Cr (III) was significantly promoted and a highly efficient removal of Cr (VI) was achieved. Under optimal operating conditions (positive bias 1 V, negative bias 2.5 V, duty ratio 20%, frequency 400 Hz, solution pH = 2), the asymmetric AC electrochemistry based on Ami-CF can achieve fast (30 s) and efficient removal (>99.11%) for 0.5–100 mg·L^−1^ Cr (VI) with a high flux of 300 L h^−1^ m^−2^. At the same time, the durability test verified the sustainability of the AC electrochemical method. For Cr (VI)-polluted wastewater with an initial concentration of 50 mg·L^−1^, the effluent concentration could still reach drinking water grade (<0.05 mg·L^−1^) after 10 cycling experiments. This study provides an innovative approach for the rapid, green and efficient removal of Cr (VI) containing wastewater at low and medium concentrations.

## 1. Introduction

Global water pollution and water shortage are key challenges for human society in the 21st century. Among them, is a large amount of chromium-polluted wastewater produced from metal plating, leather manufacturing, textile dyeing and other industries, which seriously threatens human and environmental health [1,2,3]. In the natural environment, chromium exists mainly as trivalent chromium (Cr (III)) and hexavalent chromium (Cr (VI)) [4]. Cr (III) is a micronutrient element that maintains the normal physiological activities of organisms and is a common form in nature [5]. When chromium exists in hexavalent oxygen-containing anions (e.g., Cr_2_O_7_^2−^, CrO_4_^2−^, HCrO_4_^−^) [6], it can cause cell damage in low concentrations and cause skin and stomach allergies as a result of short-term exposure, as well as liver, kidney and nervous tissue damage as a result of long-term exposure [3,7]. The World Health Organization (WHO) has set the maximum allowable level of Cr (VI) in drinking water as 0.05 mg·L^−1^ [8], and the Ministry of Ecology and Environment of China has stipulated that the content of Cr (VI) in treated domestic sewage should not exceed 0.2 mg·L^−1^ [9]. Therefore, developing efficient Cr (VI) removal technology in polluted water has become an area of great interest.

In general, Cr (VI) is highly mobile and soluble in a wide pH range, increasing its migration and potential harm, whereas Cr (III) has about 500–1000-times lower toxicity and mobility than Cr (VI) and is very easy to be removed by precipitation and adsorption [10]. Therefore, reducing Cr (VI) to Cr (III) is one of the important ways to treat chrome-containing wastewater. Although the traditional adsorption method has the advantages of low cost and simple operation, it is difficult to use on a large scale because the pores of adsorbents (such as activated carbon, zeolite, resin, etc.) are easy to plug, and the regeneration efficiency is low [11]. Recently, advanced sorbents such as porous carbon [12], graphene-based nanomaterials [13], and a metal–organic framework [14] were developed. They exhibited good adsorption performance; however, complicated fabrication processes and high cost hindered their application. Moreover, Cr (VI) removal via adsorption is only phase transfer, high toxic Cr (VI) was not detoxified to Cr (III). Although chemical reduction can effectively reduce and remove medium/high concentration Cr (VI) in wastewater, continuous use of reducing agents (such as FeSO_4_, Na_2_S_2_O_5_ and HS_2_, etc.) and hydroxides (such as NaOH, KOH, and Ca(OH)_2_, etc.) will produce a large amount of Cr-containing sludge and high alkaline solution, resulting in potential secondary pollution [15]. In contrast, electrochemical technology has the advantages of no additional chemical reagents, mild reaction conditions, simple operation and high efficiency [16,17,18]. Traditional electrochemical methods mediated by direct current (DC) systems include electrocoagulation [18], electrodialysis [19], electrodeionization [20] and electrochemical oxidation [21]/reduction [22] for sacrificial anodes (such as Fe and Al). However, due to the action of coulomb force, the cathode will repel the negatively charged hexavalent chromium containing oxygen (e.g., Cr_2_O_7_^2−^ and HCrO_4_^−^) so it cannot be effectively reduced on the electrode surface, which decreases the reduction and removal efficiency of Cr (VI). At the same time, water cracking on the electrode surface leads to a large amount of energy loss, which is a common problem in electrochemical methods [23]. In addition, parallel electrodes (iron plate, graphite plate, etc.) in traditional electrochemical reactors lack effective active sites, which is not conducive to the convective diffusion of Cr (VI) to the electrode, often showing low current efficiency and high energy consumption, and it is difficult to achieve rapid and effective removal of Cr (VI) [24]. The development of electrodes that possess interconnected macropores, high conductivity, and abundant active sites is necessary to enhance both mass transfer and current efficiency. Whereas DC can only adjust the voltage (current) to regulate the electrochemical reaction, asymmetric pulsed square-wave alternating current (AC) can achieve accurate electrode interface reaction regulation because it has four parameters: frequency, duty ratio and positive and negative bias. It has shown great advantages in electrodeposition (electroplating) [25], lithium extraction from seawater [26] and remediation of soils contaminated by heavy metals [27]. For example, Yue et al. [17] successfully recovered a large amount of Pb from wastewater using chitosan modified carbon felt electrode based on an AC electrochemical system. Lu et al. [27] designed an asymmetric AC electrochemical system-mediated soil remediation technology to achieve sustainable remediation of soil contaminated by complex heavy metals (Cu^2+^, Zn^2+^, Pb^2+^, Cd^2+^). However, there is no research on the application of AC electrochemical technology to Cr (VI) removal from wastewater.

Herein, amidoxime modified carbon felt electrode (Ami-CF) was prepared, and a novel penetrating asymmetric AC electrochemical system was constructed as a research platform to reveal an AC electrochemistry-mediated Cr (VI) removal mechanism. Due to the existence of amidoxime groups, Ami-CF is very hydrophilic and could make full use of the high surface area of the electrode. Meanwhile, amidoxime groups on Ami-CF provide strong chelating sites that can bind Cr (VI), resulting Ami-CF a 2.1–5.5 times saturated adsorption capacity than other adsorbents reported in the literature. The influences of applied voltage, duty ratio, initial Cr (VI) concentration, pH, flow rate and other coexisting ions on Cr (VI) removal by asymmetric AC electrochemical system were investigated and discussed. By employing Ami-CF as working electrode and coupled with high frequent cathode–anode conversion, AC electrochemistry not only enhances the mass transfer process while reducing side reactions, but also periodically attracts Cr (VI) at the active site under positive bias and reduces Cr (VI) to Cr (III) and repels Cr (III) under negative bias, thereby releasing the active site and constantly regenerating the Ami-CF. Under optimal operating conditions, the asymmetric AC electrochemical system based on Ami-CF can achieve fast (30 s) and efficient removal (>99.11%) for wastewater with a wide concentration (0.5–100 mg·L^−1^) of Cr (VI) with a high flux of 300 L h^−1^ m^−2^, which is superior to other methods reported. Furthermore, the removal mechanism of Cr (VI) in AC electrochemical system was thoroughly discussed combined with advanced characterizations. This study provides a new idea for the treatment of Cr (VI) containing wastewater by AC electrochemical technology in the future.

## 2. Materials and Methods

### 2.1. Chemical and Materials

Carbon felt (CF020, thickness 2 mm), purchased from Carbon Energy Technology Co., LTD. (Taiwan, China), Super P Carbon Black purchased from Alfa Aesar (Alfa Aesar, UK), polyacrylonitrile, N, N-dimethylformamide, hydroxylamine hydrochloride, sodium carbonate, dibenzoyl hydrazine, potassium dichromate, anhydrous copper sulfate, anhydrous zinc sulfate, anhydrous calcium sulfate, copper nitrate, phosphoric acid and sulfuric acid were all purchased from Alding Chemical Reagent Co., LTD (Shanghai, China). DC and AC power supply were purchased from UNI-T Co., LTD (Dongguan, China). The peristaltic pump was purchased from River Fluid Technology Co., LTD (Baoding, China). The experimental water was deionized.

### 2.2. Electrode Modification and Characterization

The original carbon felt (O-CF) was cut into discs with a diameter of 1.0 cm, and then polyacrylonitrile (PAN), Super P carbon black and N, n-dimethylformamide (DMF) were mixed and stirred at a mass ratio of 1∶1∶30 for 12 h to form a uniform slurry. The PAN-CF electrode was prepared by dipping the round carbon felt sheet with slurry and drying it in the oven (70 ℃). Then, the dried PAN-CF was put into a water bath at 70 ℃ (100 mL), and 8 g hydroxylamine hydrochloride and 6 g sodium carbonate were added to the water bath successively for hydroxylamine reaction (90 min). After the reaction, the carbon felt sheet was washed with deionized water and dried in a vacuum furnace (80 ℃) to obtain an amidoxime functionalized electrode (Ami-CF).

The electrode surface morphology was characterized by scanning electron microscopy (SEM Hitachi Regulus 8100, Tokyo, Japan). Surface functional groups were determined by Fourier to transform infrared spectroscopy (FTIR, Nicolet 6700, Thermo Scientific, Waltham, MA, USA) with a scanning range of 400 to 4000 cm^−1^ and a scanning accuracy of 2 cm^−1^. The surface chemical properties of the electrodes were analyzed by X-ray photoelectron spectroscopy (XPS, EscaLab 250Xi, Thermo Fisher Scientific, Waltham, MA, USA).

### 2.3. Batch Adsorption Experiments for Cr (VI)

Adsorption experiments were conducted using a batch approach as described in our previous studies [28,29]. The centrifuge tubes were placed in the centrifuge tubes containing 50 mL Cr (VI) solution with an initial concentration of 50 mg·L^−1^ (pH = 6 ± 0.05). A total of 0.6 mL equal samples were taken at the time points 0 min, 10 min, 30 min, 1 h, 3 h, 6 h, 12 h, 24 h, 36 h, 48 h, 60 h and 72 h, respectively. Samples were filtered and diluted properly before analysis of final Cr (VI) concentration. Each data point, including blanks (without O-CF, PAN-CF, and Ami-CF), was run in triplicate. The quasi-first-order kinetic equation (Equation (1)) and the quasi-second-order kinetic equation (Equation (2)) were used to fit the adsorption kinetic data, respectively. The formula is as follows [4,28,30]:

*Q*_t_ = *Q*_e_ [1 − exp(−*K*_1_t)] (1)

*Q*_t_ = *K*_2_t*Q*_e_^2^/(1 + *K*_2_ t*Q*_e_) (2)

where, *Q*_e_ and *Q*_t_ are the adsorption capacity (mg·g^−1^) of Cr (VI) on the electrode at adsorption equilibrium and at adsorption time t, respectively. Reaction time is t (h); The adsorption rate constants of *K*_1_ and *K*_2_ for the quasi-first order and quasi-second order kinetics, respectively.

For adsorption isotherm, 20~25 mg O-CF, PAN-CF, and Ami-CF were weighed and placed in the centrifuge tubes, 50 mL of 0 mg·L^−1^, 0.5 mg·L^−1^, 1 mg·L^−1^, 2.5 mg·L^−1^, 5 mg·L^−1^, 10 mg·L^−1^, 25 mg·L^−1^, 50 mg·L^−1^, 100 mg·L^−1^, 250 mg·L^−1^, 500 mg·L^−1^ and 1000 mg·L^−1^ Cr (VI) solution (pH = 6 ± 0.05) were added afterwards. Then the centrifuge tubes were placed in the water bath thermostatic oscillator and agitated under the same condition as described above. The adsorption time was 36 h (equilibrium time) obtained in the kinetic experiment. After the adsorption is completed, 0.6 mL equal samples are respectively taken for the determination of Cr (VI) content, calculation of adsorption capacity and removal rate. The Langmuir model (Equation (3)) and the Freundlich model (Equation (4)) are used to fit the adsorption isotherm data [28,31,32]. The formula is as follows:

*Q*_e_ = *Q*_m_*K*_L_*C*_e_/(1 + *K*_L_*C*_e_) (3)

*Q*_e_ = *K*_F_*C*_e_^N^ (4)

Where, *C*_e_ is the equilibrium concentration (mg·L^−1^), *Q*_e_ is the equilibrium adsorption capacity (mg·g^−1^), *Q*_m_ is the maximum adsorption capacity (mg·g^−1^), *K*_L_ is the Langmuir constant (L·mg^−1^), *K*_F_ is the Freundlich adsorption coefficient (mg·g^−1^·L^1/n^·mg^−1/n^). N is a heterogeneous factor.

### 2.4. Electrochemical Experiments

**Figure 1:**
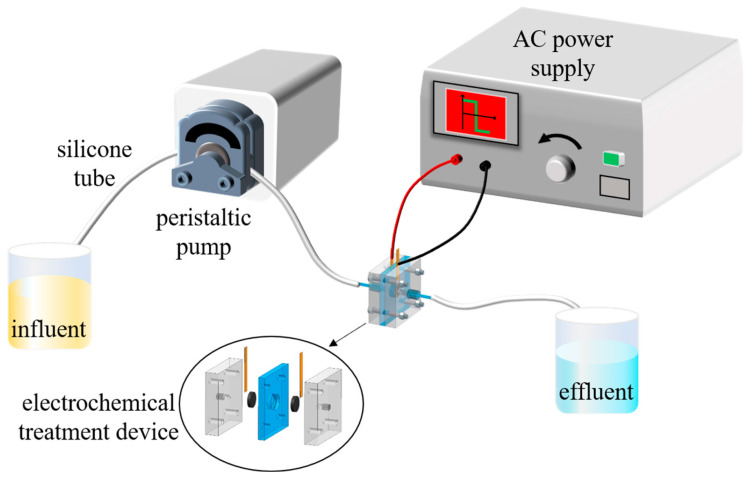
Flow-through electric treatment device based on the asymmetric alternating current system.

As shown in Figure 1, the penetrating electrochemical treatment device in the system for the treatment of wastewater containing Cr (VI) by asymmetric AC electrochemical reduction is composed of organic glass plates with a "sandwich" structure. Three plexiglass plates are fixed by four stainless steel screws, and silicone gaskets are placed between the plexiglass plates to increase airtightness and prevent water leakage; the central position of the plexiglass plate in the middle layer is provided with a groove for parallel placement of the functional carbon felt electrode. The distance between the anode and the cathode is 5 mm. The carbon felt electrode is connected to the AC power supply (Youlide UTG2025A) through the copper sheet. The plexiglass panels on the left and right sides have interfaces for connecting the liquid inlet bottle and the liquid outlet bottle through the silicone tube. During the experiment (at room temperature), 50 mL Cr (VI) solution is filled in the liquid inlet bottle, and the appropriate flow rate (mL·min^−1^) is adjusted through the peristaltic pump. The solution slowly flows to the electrochemical processing device through the silicone tube. Appropriate AC power parameters (bias, duty ratio, etc.) are set. Cr (VI) is finally reduced to Cr (III) and flows into the discharge bottle. 

### 2.5. Analysis Method

Cr (VI) was determined and analyzed by dibenzoyl dihydrazine spectrophotometry (GB 7467-87). Total chromium was determined and analyzed by ICP-OES [9]. The adsorption capacity *Q*_e_ of O-CF, PAN-CF and Ami-CF on Cr (VI) is calculated according to Equation (5). The removal rate (*R*) of Cr (VI) is calculated according to Equation (6).

*Q*_e_ = (*C*_0_ − *C*_t_)V/M (5)

*R* = [(*C*_0_ − *C*_t_)/*C*_0_] × 100% (6)

where, *C*_0_ refers to the initial concentration of Cr (VI) (mg·L^−1^), *C*_t_ refers to the concentration of Cr (VI) at moment t (mg·L^−1^), V refers to the volume of solution (L) and M refers to the mass of carbon felt electrode (g).

All experimental data were expressed as an average of three replicates with standard deviation. To compare the pollutant removal efficiencies of different treatment strategies; statistical analyses were performed through the statistical program SPSS Statistics GradPack (IBM Inc., Chicago, USA), including analysis of variance, Bartlett’s and Levine’s tests for homogeneity of variance and normality. Differences between individual means were identified using Tukey HSD-procedure at the 5% significance level. Tamhane’s T2 was selected for that equal variance between groups was not assumed.

## 3. Results and Discussion

### 3.1. Electrode Characterization

**Figure 2:**
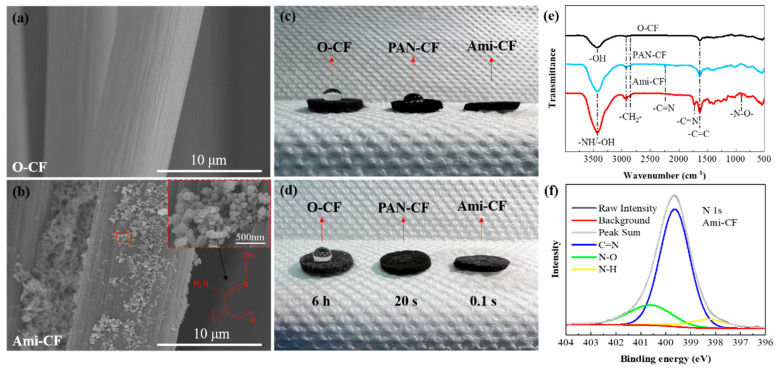
SEM images of (**a**) O-CF and (**b**) Ami-CF, (**c**,**d**) Comparison of hydrophilicity of O-CF, PAN-CF and Ami-CF, (**e**) FTIR images of O-CF, PAN-CF and Ami-CF, (**f**) XPS image of Ami-CF.

SEM characterization showed that both O-CF and Ami-CF were composed of fibers with a diameter of about 10 μm (Figure 2a,b). In contrast to the smooth surface of O-CF, the Ami-CF surface is evenly coated with a film made of carbon black nanoparticles and a polymer. Figure 2c,d) compares the hydrophilicity/hydrophobicity of O-CF, PAN-CF and Ami-CF. O-CF can trap water droplets stably on the surface for more than 6 h, PAN-CF is soaked by water droplets after 20 s, and Ami-CF is wet at the moment of contact with water droplets (less than 0.1 s). This is because the hydroxylamine reaction converts the PAN’s nitrile group (-CN) into amidoxime. Ami-CF electrode is more hydrophilic than O-CF and Pan-CF electrode due to the rich functional groups of O and N (amidoxime functionalization), which is conducive to the diffusion and reaction of Cr (VI) to the effective action site on the electrode surface in aqueous solution [33]. FTIR results confirmed the presence of amidoxime-group functional groups on the surface of Ami-CF after the hydroxylamine reaction (Figure 2e). The characteristic peaks at 2924 cm^−1^ and 2854 cm^−1^ are due to the symmetric and asymmetric stretching of -CH_2−_ on the O-CF surface [27]. The appearance of -C≡N at 2240 cm^−1^ indicates that PAN is coated in CF before the hydroxylamine reaction, and -C≡N disappears after the hydroxylamine reaction. Meanwhile, peaks at 3100–3500 cm^−1^, 1641 cm^−1^ and 903 cm^−1^, respectively, represent -NH/-OH, -C=N-, and N-O in amidoxime [34], verifying the transformation of the nitrile group to amidoxime group after hydroxylamine reaction. XPS results further confirmed the existence of an amidoxime group on the surface of Ami-CF. It can be clearly seen that Ami-CF has a strong N 1s peak (Figure 2f), and further peak segmentation results show N-H (398.3 eV), C=N (399.7 eV) and N-O (400.7 eV) groups [33].

### 3.2. Adsorption Kinetics and Adsorption Isotherms

**Figure 3:**
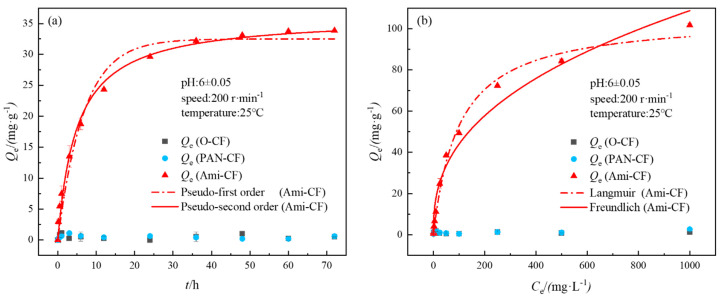
Adsorption kinetics (**a**) and adsorption isotherms (**b**) of Cr (VI) onto different carbon felts.

The adsorption kinetics and isothermal adsorption process of Cr (VI) on carbon felt electrode material are shown in Figure 3a,b. It can be seen that under the condition that the initial Cr (VI) concentration is 50 mg·L^−1^, the adsorption capacities of O-CF, PAN-CF and Ami-CF on Cr (VI) show obvious differences with the change of adsorption time. The adsorption of Cr (VI) on Ami-CF is a typical kinetic process. The adsorption capacity increases rapidly within the first 12 h, and the adsorption equilibrium is reached in about 36 h (36 h is taken as the adsorption equilibrium time in subsequent isothermal adsorption experiments), and the highest adsorption capacity is 33.86 mg·g^−1^. It was much higher than the equilibrium adsorption capacity of O-CF and PAN-CF (~1.1 mg·g^−1^). Table S1 lists the fitting parameters of the quasi-first-order and quasi-second-order kinetic models for the adsorption kinetics of Cr (VI) by Ami-CF, and the equation fitting coefficients R^2^ are 0.976 and 0.993, respectively. The pseudo-first-order kinetics are based on the assumption that the adsorption rate is controlled by the diffusion step and the pseudo-second-order kinetics are based on the assumption that the adsorption rate is controlled by the chemisorption mechanism [35,36,37]. Therefore, the quasi second-order kinetic equation can better describe the adsorption process of Cr (VI) by Ami-CF, indicating that the adsorption process is mainly controlled by chemical adsorption [32].

Langmuir and Freundlich isotherm models are often used to reveal the interaction mechanism between heavy metal ions and adsorbents. Among them, the Langmuir model assumes that metal ions occur at a uniform interface through monolayer deposition. In contrast, the Freundlich isotherm model describes adsorption at a non-uniform interface [32]. The adsorption isotherms of Cr (VI) on different carbon felt electrodes are shown in Figure 3b. Due to the lack of effective adsorption sites, the saturated adsorption capacity of O-CF and PAN-CF on Cr (VI) is only 1.99–2.71 mg·g^−1^, whereas that of the Ami-CF-containing amidoxime group on Cr (VI) can reach 101.73 mg·g^−1^. It is 2.1–5.5 times the saturated adsorption capacity of biochar, oxidized composite materials and other adsorbents reported in the literature (Table S3) [30,38,39]. The fitting coefficient R^2^ of the Langmuir model for Ami-CF adsorption of Cr (VI) is 0.992, whereas that of the Freundlich model is 0.968 (Table S2). Therefore, the Langmuir model is more suitable for describing Ami-CF adsorption of Cr (VI). This indicates that the adsorption behavior of Cr (VI) on Ami-CF is mainly a single-layer adsorption reaction [34]. The infinitesimal number *R*_L_ is commonly used to judge whether the adsorption process is conducive to its occurrence, and its expression is shown in Equation (7).

*R*_L_ = 1/(1 + *K*_L_ *C*_0_) (7)

When *R*_L_ is 0–1, it indicates favorable adsorption, *R*_L_ > 1 indicates unfavorable adsorption, *R*_L_ = 1 indicates linear adsorption, and *R*_L_ = 0 indicates irreversible adsorption [4]. The results show that the *R*_L_ value ranges from 0.08 to 0.9, which indicates that Ami-CF is favorable for the adsorption of Cr (VI). In addition, with the increase in initial Cr (VI) concentration, *R*_L_ value decreases continuously, indicating that the higher the concentration of pollutants, the more favorable the adsorption.

### 3.3. Factors Influencing the Removal Efficiency of Cr (VI)

In the traditional DC-mediated treatment of wastewater containing heavy metals, parameter optimization is limited to the difference of control voltage, whereas in the AC mediated treatment, the regulated parameters generally include positive and negative bias, frequency and duty ratio. The preliminary experimental results show that when the AC frequency is controlled at 400 Hz, the side reaction of electrolytic water is minimal. Therefore, the square-wave AC frequency was controlled to be 400 Hz, and the influences of positive and negative bias, duty cycle, solution flow rate, initial solution pH, initial concentration of Cr (VI) and other coexisting ions on Cr (VI) removal by AC electrochemical system were investigated. Two parallel treatments were set for each group of experiments.

#### 3.3.1. Effects of Bias, Duty Cycle and Flow Velocity

**Figure 4:**
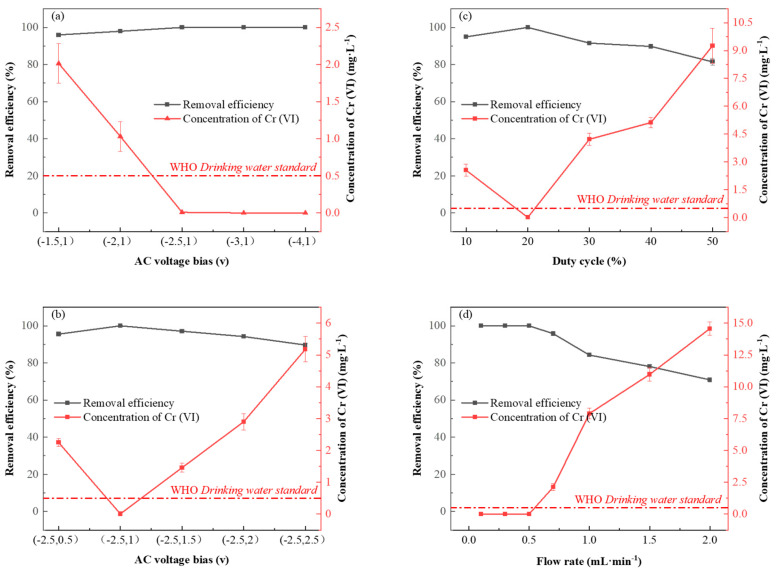
(**a**) Comparison of removal rate under different negative bias. (**b**) Comparison of removal rate under different positive bias. (**c**) Comparison of removal rate under different duty cycle. (**d**) Comparison of removal rate at different flow rates.

An AC electrochemical system removes Cr (VI) in solution by applying asymmetric square wave bias. The volume of Cr (VI) solution (50 mL), solution pH (2 ± 0.05), a flow rate of a peristaltic pump (0.5 mL·min^−1^) and duty ratio (20%) in the liquid inlet bottle were kept unchanged, and the positive bias was fixed at 1V. The reduction and removal efficiency of Cr (VI) by AC electrochemical system under a negative bias voltage of −1.5 V, −2 V, −2.5 V, −3 V and −4 V were studied successively. The results are shown in Figure 4a; the removal rate of Cr (VI) increases gradually with the increase in negative bias voltage, which may be because the increase in applied potential accelerates the mass transfer and electron transfer of Cr (VI) in solution, which is conducive to the reduction of Cr (VI) on the electrode surface. When the negative bias increases from −1.5 V to −2.5 V, the removal rate of Cr (VI) increases from 95.97% to 99.98%, and the mean residual concentration of Cr (VI) is 8.33 μg·L^−1^, which meets the limit value of Cr (VI) of the national sanitary standard for drinking water (<0.05 mg·L^−1^). When a negative bias is further increased, the residual concentration of Cr (VI) remains below 4 μg·L^−1^. Therefore, −2.5 V is the best voltage for negative bias in the subsequent experiments. Similarly, by fixing negative bias as −2.5 V, the electrochemical removal effects of positive bias 0.5 V, 1 V, 1.5 V, 2 V and 2.5 V on Cr (VI) are studied successively. As shown in Figure 4b, with positive bias increasing from 0.5 V to 2.5 V, the removal rate of Cr (VI) presents a trend of first increasing and then decreasing. This may be attributed to the Coulomb repulsion effect, which inhibits the electrochemical reduction efficiency of Cr (VI) when the positive bias voltage is too low. However, when the positive bias voltage is too high, the reduced Cr (III) may be re-oxidized into Cr (VI), and the removal rate of Cr (VI) will eventually decline. Therefore, negative bias voltage −2.5 V and positive bias voltage 1 V were selected in the subsequent treatment experiment to ensure the best removal efficiency.

The duty cycle refers to the ratio of the high-level (positive bias) duration in a cycle. Other conditions were controlled in accordance with the above studies. The duty ratio was controlled as 10%, 20%, 30%, 40% and 50% separately to study its influence on the electrochemical reduction of Cr (VI). As shown in Figure 4c, when the duty ratio increases from 10% to 20%, the removal rate of Cr (VI) increases significantly, but when the duty ratio continues to increase from 20% to 50%, the removal rate of Cr (VI) decreases significantly. This may be because too large a duty cycle inhibits the reduction of Cr (VI), ultimately leading to the decrease in the Cr (VI) removal rate. However, when the duty cycle is too low, the alternating current is similar to the DC process in which stable negative bias is applied, resulting in the coulomb repulsion of the electrocatalytic reduction of Cr (VI) [16]. When the duty cycle is 20%, a good balance can be achieved between the capture of Cr (VI) oxygen-containing anions by chelating sites on Ami-CF and the reduction of Cr (VI) to Cr (III), thus achieving the best removal effect [2].

The effect of flow rate on Cr (VI) removal by penetrating electrochemical treatment devices is shown in Figure 4d. At low flow rates (0.1 mL·min^−1^, 0.3 mL·min^−1^), Cr (VI) in the solution could be removed entirely due to the long residence time between electrodes and sufficient contact between Cr (VI) and Ami-CF surface. When the flow rates were 0.5 mL·min^−1^ and 0.7 mL·min^−1^, the removal efficiency of Cr (VI) was about 99.98% and 95.76%, respectively. When the flow rate is further increased, the higher flow rate will reduce the adsorption-reduction process of Cr (VI) on the electrode surface, decreasing removal efficiency. Therefore, the flow rate of the peristaltic pump is set as 0.5 mL·min^−1^.

#### 3.3.2. Effects of Initial Cr (VI) Concentration and Solution pH

**Figure 5:**
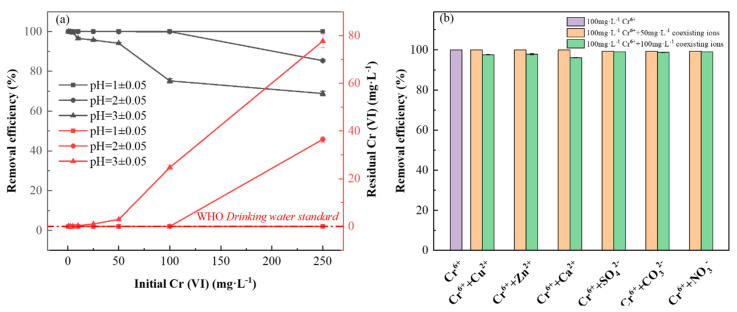
(**a**) Effect of different pH and Cr (VI) concentration on Cr (VI)removal rate. (**b**) Effect of Cu (II), Zn (II), Ca (II), SO_4_^2−^, CO_3_^2−^ and NO_3_^−^ at different concentrations on Cr (VI) removal rate of 100 mg·L^−1^.

As shown in Figure 5a, the removal effect of the asymmetric AC electrochemical system on Cr (VI) with different concentrations at different pH values has been tested. When the fluid pH was 1 ± 0.05, Cr (VI) could be completely removed in a large range (0.5–250 mg·L^−1^). When the pH of the feed solution was adjusted to 2 ± 0.05, the Cr (VI) removal rate decreased slightly. Still, it remained at a high Cr (VI) removal rate (more than 85.04%) in the range of experimental concentration (0.5–250 mg·L^−1^) and Cr (VI) in the range of 0.5–100 mg·L^−1^ could almost be completely removed. The residual Cr (VI) is lower than 0.05 mg·L^−1^, which meets the hygienic limit of drinking water. When the pH of the feeding solution is adjusted to 3 ± 0.05, the removal rate of Cr (VI) at 0.5–50 mg·L^−1^ concentration can reach more than 93.74%. 

However, when the concentration of Cr (VI) in the feed solution is too high, the removal rate obviously decreases. The main reasons for the reduction of Cr (VI) removal rate with the increase in pH are as follows: (1) According to E (electric potential)-pH diagram for chromium speciation predominance [40], the reduction of Cr (VI) to Cr (III) under acidic conditions is advantageous from the perspective of thermodynamics, because its standard potential increases with the increase in proton concentration. When pH value is ≥ 3, the standard potential of Cr (VI) reduction decreases, thus reducing the possibility of a reaction [41]; (2) when pH is 1–3, it is simulated by Visual MINTEQ 3.1 that Cr (VI) mainly exists in the form of Cr_2_O_7_^2−^ and HCrO_4_^−^ and participates in the reduction reaction from Cr (VI) to Cr (III) [6]. The reaction on the cathode is as follows: Equations (8)–(11) [42]; and (3) although Cr (III) generated by the reaction does not begin to form Cr (OH)_3_ precipitate until pH > 4 [24], the hydrogen evolution reaction equation (10) under negative potential provides a local slightly alkaline environment for the electrode, increasing local surface pH [21]. Thus, insoluble Cr (OH)_3_ films or colloids may be generated on the electrode surface (Equation (11)) [42], thus hindering electron transfer and inhibiting further reduction of Cr (VI) [43]. Although the optimal removal efficiency can be attained at pH = 1 ± 0.05, pH levels of actual chrome-containing wastewater typically range from 1 to 3, and economic considerations should also be taken into account [24,44]. Therefore, a pH of 2 ± 0.05 has been selected for the remediation of wastewater containing Cr (VI). In general, the removal effect of Cr (VI) on the AC electrochemical system decreases with the increase in initial Cr (VI) concentration and pH, which is consistent with studies in the literature [9,41]. It should be noted that the calculated flux of the electrochemical treatment device is 300 L h^−1^ m^−2^, and the contact time between Cr (VI) and Ami-CF in solution is only 30 s. Therefore, this method can achieve rapid and efficient removal effect on medium and high concentration Cr (VI) wastewater at low pH, which is superior to adsorption, precipitation, photocatalytic reduction and other methods [11,15].

Cr_2_O_7_^2−^ + 14H^+^ + 6e^−^ → 2Cr^3+^ + 7H_2_O (8)

HCrO_4_^−^ + 4H^+^+3e^−^ → Cr^3+^ + 4H_2_O (9)

2H^+^ + 2e^−^ → H_2_ (10)

Cr^3+^ + 3OH^−^ → 3Cr(OH)_3_ (11)

#### 3.3.3. Removal of Cr (VI) in Multi-Ions Solutions

Considering the complexity of actual wastewater components [44], Cu^2+^, Zn^2+^ and Ca^2+^ were taken as representative coexisting heavy metals and alkali metal cations, and SO_4_^2−^, CO_3_^2−^ and NO_3_^−^ were taken as representative anions. The influence of coexisting ions on the removal of Cr (VI) by this method was discussed. The optimized electrochemical and solution parameters were selected as the experimental conditions; namely, the AC frequency was 400 Hz, the bias voltage was (−2.5, 1) V, the duty ratio was 20%, the pH of the solution was 2 ± 0.05 and the flow rate of the peristaltic pump was 0.5 mL·min^−1^. An amount of 50 mL Cr (VI) containing 100 mg·L^−1^ and different concentrations of Cu^2+^/Zn^2+^/Ca^2+^/SO_4_^2−^/CO_3_^2−^/NO_3_^−^ solutions were treated, and two parallel treatments were set up in each group.

As shown in Figure 5b, under optimized operating parameters, the AC electrochemical system can achieve a 99.97% removal rate for Cr (VI) solution with an initial concentration of 100 mg·L^−1^. The removal rate of Cr (VI) was further improved when the solution contained 50 mg·L^−1^ co-existing Cu^2+^/Zn^2+^/Ca^2+^. This may be because the introduction of appropriate Cu^2+^/Zn^2+^/Ca^2+^ can improve the ionic strength and conductivity of the solution. The reduction potentials of Zn^2+^ and Ca^2+^ are relatively high, so they only act as electrolytes in the whole reaction system and do not participate in chemical reactions. Relatively speaking, Cu^2+^ is quickly reduced to Cu^+^/Cu^0^ and acts as a reducing agent to enhance the reduction removal of Cr (VI) [45]. However, when the concentration of Cu^2+^, Zn^2+^ and Ca^2+^ was further increased, the removal rate of Cr (VI) began to decrease. This may be because a large number of positively charged Cu^2+^, Zn^2+^ and Ca^2+^ will gather on the cathode surface through electrostatic attraction and compete with Cr (VI) for reaction sites on the electrode surface [46], resulting in a decline in the removal effect of Cr (VI). 

Similar to the enhanced conductivity of heavy metals and alkali metal ions, when the solution contains 50 mg·L^−1^ coexisting SO_4_^2−^/CO_3_^2−^/NO_3_^−^, the removal rate of Cr (VI) does not decrease, although it has no promoting effect. There are two possible reasons: (1) Compared with the reduction of S^6+^/C^4+^ and N^5+^ in SO_4_^2−^/CO_3_^2−^/NO_3_^−^, the reduction of Cr^6+^ to Cr^3+^ in HCrO_4_^−^ is more likely [47,48]; and (2) for the coexisting cations (Cu^2+^/Zn^2+^/Ca^2+^) and anions (SO_4_^2−^/CO_3_^2−^/NO_3_^−^) with the same concentration involved in this experiment, the ionic strength obtained from the conversion of anions is smaller than that of cations, and the conductivity of the solution containing the coexisting anions is relatively small. When the concentrations of SO_4_^2−^, CO_3_^2−^ and NO_3_^−^ were further increased, the removal rate of Cr (VI) decreased slightly. This may be because a large number of negatively charged SO_4_^2−^, CO_3_^2−^ and NO_3_^−^ will accumulate on the anode surface through electrostatic attraction, which inhibits the reduction of Cr^6+^ when the electrode is switched to the cathode later. In addition, Visual MINTEQ 3.1 was used to simulate the distribution of chromium species in 100 mg·L^−1^ Cr (VI) solution at pH 2. The results showed that Cr_2_O_7_^2−^ and HCrO_4_^−^ were the dominant chemical species, accounting for 6.4% and 93%, respectively. When different concentrations of Cu^2+^/Zn^2+^/Ca^2+^/SO_4_^2−^/CO_3_^2−^/NO_3_^−^ were present in the solution, the species distribution of Cr (VI) did not change significantly (Table S4). It should be note that the composition of real wastewater is definitely much more complex than that in the current experiment. For example, various cations and anions, as well as dissolved organic matters, may co-existed in the real wastewater. Thus further research should be conducted to investigate the removal performance of the AC electrochemical system for real wastewater.

#### 3.3.4. Direct/Alternating Current Electrochemical Method for Removing Cr (VI)

**Figure 6:**
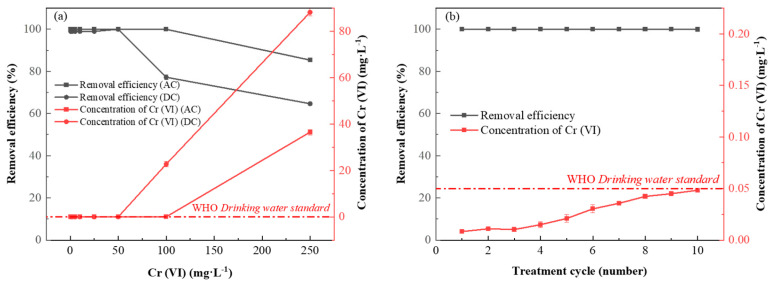
(**a**) Comparison of Cr (VI) removal by direct current (DC) and alternating current (AC) methods. (**b**) Cr (VI) removal as the function of treatment cycle by Ami-CF in the long-term experiment.

The solution conditions (pH = 2 ± 0.05, flow rate 0.5 mL·min^−1^) and negative bias voltage (DC −2.5 V, AC −2.5 V, 1 V) were consistent. The removal effects of Cr (VI) from 0.5–250 mg·L^−1^ were compared between DC and AC electrochemical methods. The results are shown in Figure 6a. For 0.5–50 mg·L^−1^ Cr (VI), 100% removal was achieved by both DC and AC methods. However, when the initial concentration of Cr (VI) increased further, the removal efficiency of the DC method decreased significantly. For example, when the infusion concentration was 100 mg·L^−1^ and 250 mg·L^−1^, the removal efficiency of the DC method was 77.22% and 64.65%, respectively. In contrast, the AC method can still maintain the removal rate of 99.97% for Cr (VI) of 100 mg·L^−1^, and even the Cr (VI) of 250 mg·L^−1^ can still reach 85.37%, which is more than 20% higher than the DC method, which further highlights the superiority of AC method.

### 3.4. Stability of Functional Electrode and Electrochemical Treatment Device

In order to test the stability of the functional electrode Ami-CF and the electrochemical treatment device, 50 mg·L^−1^ Cr (VI) solution was continuously injected into the electrochemical filtration device at the rate of 0.5 mL·min^−1^ by a peristaltic pump and 50 mL was taken as a single dose. The removal efficiency in the long-term operation process shown in Figure 6b. In a total of 10 experiments, the removal rate remained stable above 99.9%, and the Cr (VI) concentration after treatment was lower than 0.05 mg·L^−1^, which met the safety standard of Cr (VI) for drinking water stipulated by the World Health Organization. This excellent performance indicates that the functional electrode Ami-CF has excellent recycling performance, and the electrochemical treatment device can maintain excellent removal efficiency for a long time. Meanwhile, Cr (VI) was converted into Cr (III), which could be easily separated and recovered through precipitating with alkali, consequently reduced the overall cost. Thus the long time and stable performance, together with the concept of turning waste into resource, demonstrated the proposed method has broad application prospects.

### 3.5. Removal Mechanism

**Figure 7:**
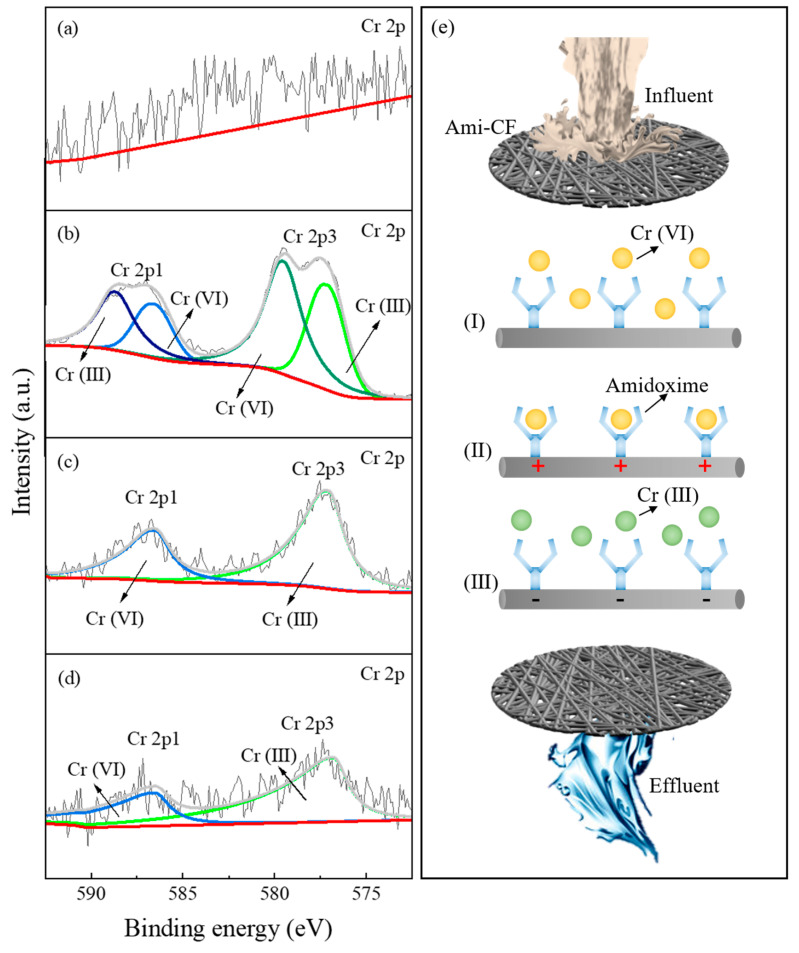
(**a**) before and (**b**) after the Cr(VI) adsorbed on Ami-CF, (**c**) DC and (**d**) AC method on Ami-CF, and (**e**) reduction process of Cr (VI) mediated by AC.

In order to explore the removal mechanism of Cr (VI) by this method, the Ami-CF after Cr (VI) adsorption, Cr (VI) treatment by direct current electrochemistry, and AC electrochemistry were characterized by XPS. After carbon correction, there was no characteristic peak of Cr on the surface of unused Ami-CF as a control group (Figure 7a). In Figure 7b, after Cr (VI) adsorption, Ami-CF shows characteristic peaks of Cr (III) 2p 1/2, Cr (VI) 2p 1/2, Cr (VI) 2p 3/2 and Cr (III) 2p 3/2 at 588.8 eV, 586.7 eV, 579.6 eV and 577.3 eV, respectively [49]. This is consistent with the adsorption results of Cr (VI) by Ami-CF (Figure 3a,b), which further confirms the strong adsorption capacity of the amidoxime group in Ami-CF on Cr (VI). In addition, the oxygen-containing functional groups (-OH, -NH) in Ami-CF can reduce Cr (VI) to Cr (III), and then adsorb it on the electrode surface [50,51]. In Figure 7c,d, Ami-CF after DC and AC treatment showed prominent characteristic peaks of Cr (VI) and Cr (III) at 586.7 eV, 586.6 eV, 577.3 eV, 577.2 eV, respectively. Therefore, it is speculated that the reduction and removal of Cr (VI) under AC mediation mainly include the following three steps (Figure 7e): In step (1), all Cr (VI) oxygen-containing anions are randomly distributed in aqueous solution without applied voltage. In step (2), when a positive bias is applied, the oxygen-containing anion of Cr (VI) migrates towards the anode in the applied electric field. It is adsorbed by the amidoxime group on the Ami-CF surface to form an electric double layer on the electrode surface. In step (3), voltage switching and cathode electron transfer reduce Cr (VI) to Cr (III) and release Cr (III) into the solution. At the same time, the previously occupied active sites can be recovered. In the subsequent reaction, new Cr (VI) oxygen-containing anions can be adsorption-fixation-reduction again to maintain the continuous process of the whole reaction.

## 4. Conclusions

In this study, we provide a stable and efficient treatment method for wastewater containing Cr (VI). To improve adsorption and subsequent eletroreduction of Cr (VI) to Cr (III), facile fabrication of Ami-CF from commercial carbon felt by functionalization with amine oxime was developed and has excellent hydrophilicity and a strong adsorption capacity for Cr (VI) with saturated adsorption capacity of 101.73 mg·g^−1^. Coupled with the Ami-CF electrode, the Coulomb repulsion effect and the side reaction of electrolytic water were suppressed under the high frequency anode and cathode conversion (asymmetric AC), and the diffusion mass transfer rate of Cr (VI) in the solution was promoted. Consequently, the asymmetric AC electrochemistry based on Ami-CF can rapidly (in 30 s) turning 0.5–100 mg·L^−1^ Cr (VI) into the safety standard of WHO drinking water with a high flux of 300 L h^−1^ m^−2^. Long-term operation of a total of 10 cycles demonstrated a high, stable and efficient removal performance. These results indicate that the asymmetric AC electrochemistry coupled with Ami-CF exhibit great application potential for the treatment of medium-to-high concentration Cr (VI)-containing wastewater. Further study is needed to scale-up the asymmetric AC electrochemical system and treat real wastewater with a much more complex composition.

## Data Availability

Not applicable.

## Supplementary Materials

The following are available online at https://www.mdpi.com/article/10.3390/nano13050952/s1. Table S1: Adsorption kinetics fitting parameters of Cr (VI) by Ami-CF; Table S2: Fitting parameters of adsorption isotherm of Cr (VI) by Ami-CF; Table S3: Adsorption capacity of adsorbents for Cr (VI); Table S4: Influence of different concentrations of Cu (II), Zn (II) and Ca (Ⅱ) on distribution percentage of the Cr (VI) species from the simulation results for 100 mg/L Cr (VI) solution at pH 2 by using Visual MINTEQ 3.1. The following references were cited in Supplementary Materials: [52,53,54,55,56,57,58,59,60].
