# Supplementary material for: Asymmetric Alternative Current Electrochemical Method Coupled with Amidoxime-Functionalized Carbon Felt Electrode for Fast and Efficient Removal of Hexavalent Chromium from Wastewater
Source: Nanomaterials (Basel). 2023 Mar 6;13(5):952. doi: 10.3390/nano13050952 (PMC10005244; doi:10.3390/nano13050952)
Supplement: Supplementary file 1 [file nanomaterials-13-00952-s001.zip › nanomaterials-2209567-supplementary.pdf]

nanomaterials MDPI

Supporting Information

Asymmetric Alternative Current Electrochemical Method Coupled with Amidoxime-Functionalized Carbon Felt Electrode for Fast and Efficient Removal of Hexavalent Chromium from Wastewater

Yunze Yang [1], Lun Lu [2,*], Yi Shen [3], Jun Wang [4], Liangzhong Li [2], Ruixue Ma [2], Zahid Ullah [5], Mingdeng Xiang [2], Yunjiang Yu [1,2,*]

[1] Key Laboratory of Subsurface Hydrology and Ecological Effects in Arid Region, Ministry of Education, School of Water and Environment, Chang'an University, Xi'an, 710064, China; 2020129058@chd.edu.cn
[2] State Environmental Protection Key Laboratory of Environmental Pollution Health Risk Assessment, South China Institute of Environmental Sciences, Ministry of Ecology and Environment, Guangzhou, 510655, China; liliangzhong@scies.org (L.L.), maruixue@scies.org (R.M.), xiangmingdeng@scies.org (M.X.)
[3] Key Laboratory of Microbial Technology for Industrial Pollution Control of Zhejiang Province, College of Environment, Zhejiang University of Technology, Hangzhou 310032, China; shenyi@zjut.edu.cn
[4] State Key Laboratory of Separation Membranes and Membrane Processes, School of Environmental Science and Engineering, Tiangong University, Tianjin 300387, China; jun.wang@tiangong.edu.cn
[5] State Key Laboratory of Biogeology and Environmental Geology, School of Environmental Studies, China University of Geosciences, Wuhan 430074, China; 2201890048@cug.edu.cn
* Correspondence: lulun@scies.org (L.L.), yuyunjiang@scies.org (Y.Y.)

Table S1. Adsorption kinetics fitting parameters of Cr (VI) by Ami-CF

Models	Parameters	Values
Pseudo-first order model	$Q_{e,\,exp}$/mg·g^{-1}	33.86
	$Q_{e,\,cal}$/mg·g^{-1}	32.50
	K_1/h^{-1}	0.1486
	R^2	0.976
Pseudo-second order model	$Q_{e,\,exp}$/mg·g^{-1}	33.86
	$Q_{e,\,cal}$/mg·g^{-1}	36.13
	K_2/g·(mg·h)$^{-1}$	0.0056
	R^2	0.993

Table S2. Fitting parameters of adsorption isotherm of Cr (VI) by Ami-CF

Models	Parameters	Values
Langmuir	Q_m/mg·g^{-1}	105.79
	K_L/L·mg^{-1}	0.01
	R^2	0.992
Freundlich	N	0.39
	K_F/ mg·g^{-1}·L$^{1/n}$·mg$^{-1/n}$	7.18
	R^2	0.968

Table S3. Adsorption capacity of adsorbents for Cr (VI)

Adsorbent	pH	Adsorption capacity	References
Mn-incorporated ferrihydrite	5.0	48.5 mg·g^{-1}	[1]
TOCNF Grafted With PABS Copolymer	3.0	5.263 mg·g^{-1}	[2]
Poultry manure-derived biochar	4.0	19.09 mg·g^{-1}	[3]

Polyaniline nanowires-coated polypropylene filter	7	66.7 $mg{\cdot}g^{-1}$	[4]
$ZnFe_2O_4$	2.5	34.25 $mg{\cdot}g^{-1}$	[5]
Straw and sludge-based activated carbon	6.0	15 $mg{\cdot}g^{-1}$	[6]
Fe-Al hydroxides	5.6	20 $mg{\cdot}g^{-1}$	[7]
Chlorapatite	7.0	10 $mg{\cdot}g^{-1}$	[8]
Carbon nano-onions	6.0	60 $mg{\cdot}g^{-1}$	[9]
Tobacco petiole Pyrolytic biochar	6.0	40 $mg{\cdot}g^{-1}$	[10]

Table S4. Influence of different concentrations of Cu (II), Zn (II) and Ca (II) on distribution percentage of the Cr (VI) species from the simulation results for 100 mg/L Cr (VI) solution at pH 2 by using Visual MINTEQ 3.1.

**Cr (VI) Species**	**50/100 $mg{\cdot}L^{-1}$ Cu^{2+} (%)**	**50/100 $mg{\cdot}L^{-1}$ Zn^{2+} (%)**	**50/100 $mg{\cdot}L^{-1}$ Ca^{2+} (%)**	**50/100 $mg{\cdot}L^{-1}$ SO_4^{2-} (%)**	**50/100 $mg{\cdot}L^{-1}$ CO_3^{2-} (%)**	**50/100 $mg{\cdot}L^{-1}$ NO_3^- (%)**
$Cr_2O_7^{2-}$	6.5/6.6	6.5/6.6	6.5/6.6	6.5/6.5	6.4/6.4	6.5/6.5
$HCrO_4^-$	92.9/92.8	92.9/92.9	92.9/92.9	93/92.9	93/93	93/93
H_2CrO_4 (aq)	0.5/0.5	0.5/0.5	0.5/0.5	0.5/0.5	0.5/0.5	0.5/0.5

References

1. Liang, C.; Fu, F.; Tang, B. Mn-incorporated ferrihydrite for Cr(VI) immobilization: Adsorption behavior and the fate of Cr(VI) during aging. *J Hazard Mater* **2021**, *417*, 126073.http://doi.org/10.1016/j.jhazmat.2021.126073.
2. Yu, Y.H.; An, L.; Bae, J.H.; Heo, J.W.; Chen, J.; Jeong, H.; Kim, Y.S. A Novel Biosorbent From Hardwood Cellulose Nanofibrils Grafted With Poly(m-Aminobenzene Sulfonate) for Adsorption of Cr(VI). *Front Bioeng Biotechnol* **2021**, *9*, 682070.http://doi.org/10.3389/fbioe.2021.682070.
3. Ghani, U.; Jiang, W.; Hina, K.; Idrees, A.; Iqbal, M.; Ibrahim, M.; Saeed, R.; Irshad, M.K.; Aslam, I. Adsorption of Methyl Orange and Cr (VI) Onto Poultry Manure-Derived Biochar From Aqueous Solution. *Front Environ Sci* **2022**, *10*.http://doi.org/10.3389/fenvs.2022.887425.
4. Pi, S.-Y.; Wang, Y.; Pu, C.; Mao, X.; Liu, G.-L.; Wu, H.-M.; Liu, H. Cr(VI) reduction coupled with Cr(III) adsorption/ precipitation for Cr(VI) removal at near neutral pHs by polyaniline nanowires-coated polypropylene filters. *J Taiwan Inst Chem Eng* **2021**, *123*, 166−174.http://doi.org/10.1016/j.jtice.2021.05.019.
5. Jia, Z.; Qin, Q.; Liu, J.; Shi, H.; Zhang, X.; Hu, R.; Li, S.; Zhu, R. The synthesis of hierarchical $ZnFe_2O_4$ architecture and their application for Cr(VI) adsorption removal from aqueous solution. *Superlattices Microstruct* **2015**, *82*, 174−187.http://doi.org/10.1016/j.spmi.2015.01.028.
6. Fan, L.; Wan, W.; Wang, X.; Cai, J.; Chen, F.; Chen, W.; Ji, L.; Luo, H.; Cheng, L. Adsorption Removal of Cr(VI) with Activated Carbon Prepared by Co-pyrolysis of Rice Straw and Sewage Sludge with $ZnCl_2$ Activation. *Water Air Soil Pollut* **2019**, *230*.http://doi.org/10.1007/s11270-019-4305-8.
7. Wang, X.-H.; Liu, F.-F.; Lu, L.; Yang, S.; Zhao, Y.; Sun, L.-B.; Wang, S.-G. Individual and competitive adsorption of Cr(VI) and phosphate onto synthetic Fe-Al hydroxides. *"Colloids Surf, A"* **2013**, *423*, 42-49.http://doi.org/10.1016/j.colsurfa.2013.01.026.
8. Han, X.; Zhang, Y.; Zheng, C.; Yu, X.; Li, S.; Wei, W. Enhanced Cr(VI) removal from water using a green synthesized nanocrystalline chlorapatite: Physicochemical interpretations and fixed-bed column mathematical model study. *Chemosphere* **2021**, *264*.http://doi.org/10.1016/j.chemosphere.2020.128421.
9. Sakulthaew, C.; Chokejaroenrat, C.; Poapolathep, A.; Satapanajaru, T.; Poapolathep, S. Hexavalent chromium adsorption from aqueous solution using carbon nano-onions (CNOs). *Chemosphere* **2017**, *184*, 1168−1174.http://doi.org/10.1016/j.chemosphere.2017.06.094.
10. Zhang, X.; Fu, W.; Yin, Y.; Chen, Z.; Qiu, R.; Simonnot, M.-O.; Wang, X. Adsorption-reduction removal of Cr(VI) by tobacco petiole pyrolytic biochar: Batch experiment, kinetic and mechanism studies. *Bioresour Technol* **2018**, *268*, 149−157.http://doi.org/10.1016/j.biortech.2018.07.125.
